# Prenatal diagnosis of Bardet–Biedl syndrome in a case of hyperechogenic kidneys: Clinical use of DNA sequencing

**DOI:** 10.1002/ccr3.859

**Published:** 2017-03-02

**Authors:** Santiago Garcia‐Tizon Larroca, Vangeliya Blagoeva Atanasova, Maria Orera Clemente, Anna Aluja Mendez, Virginia Ortega Abad, Ricardo Perez Fernandez‐Pacheco, Juan De León Luis, Francisco Gamez Alderete

**Affiliations:** ^1^ Fetal medicine unitDepartment of Obstetrics and GynaecologyHospital General Universitario Gregorio MarañonMadridSpain

**Keywords:** Bardet–Biedl syndrome, hyperechogenic kidneys, prenatal diagnosis

## Abstract

Bardet–Biedl syndrome (BBS) is a ciliopathy that is responsible for multiple visceral abnormalities. This disorder is defined by a combination of clinical signs, many of which appear after several years of development. BBS may be suspected antenatally based on routine ultrasound findings of enlarged hyperechogenic kidneys and postaxial polydactyly.

## Introduction

Bardet–Biedl syndrome (BBS) is a ciliopathic human genetic disorder that causes multiple visceral abnormalities such as obesity, postaxial polydactyly, pigmentary retinopathy, neurological movement disorders, and genitourinary anomalies 1. Its prevalence ranges from 1/125,000 to 1/175,000. Other specific clinical features may include ataxia/poor coordination, learning disabilities, mild hypertonia, hypertension, diabetes mellitus, hypodontia, anosmia, auditory deficiencies, hepatic fibrosis, and Hirschsprung disease. Individual clinical phenotypes are highly variable.

Extensive research during the last decade has contributed to the understanding of the genetic substrate 2, 3, 4 and pathophysiology of ciliary disorders 5, 6.

Ciliopathies are manifested in multiple clinical conditions characterized by defects of the cilium and its gripping structure, the basal body. Inheritance is mainly autosomal recessive and can result from mutations of different genes known to play important roles in cilia, structures that project from the surfaces of cells 7.

Bardet–Biedl syndrome mutations are usually detected via genomic sequencing of the coding regions of their causative genes. Karyotyping and array comparative genomic hybridization are not routinely utilized because genomic rearrangements and large deletions are uncommon 8. Readily available sequencing panels allow screening of multiple BBS genes with a sensitivity of 70% 9.

## Case Presentation

A 33‐year‐old gravida had her first pregnancy after IVF treatment and embryo transfer. The first trimester combined screening revealed a low risk of aneuploidies. Her parents were not consanguineous and did not have any medical condition or family history of inherited diseases or renal dysfunction.

**Figure 1:**
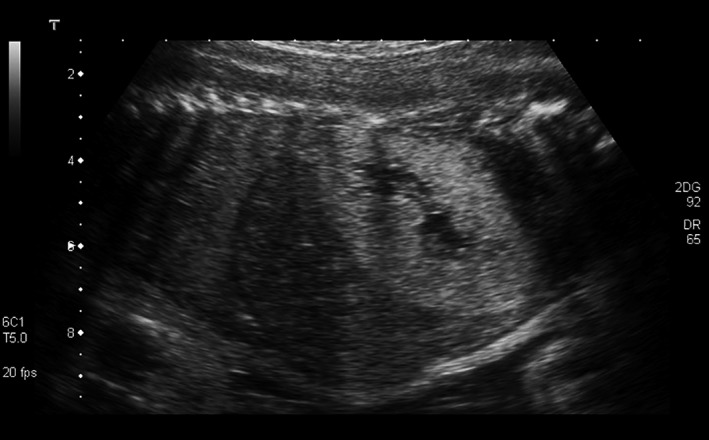
Hyperechoic kidney.

**Figure 2:**
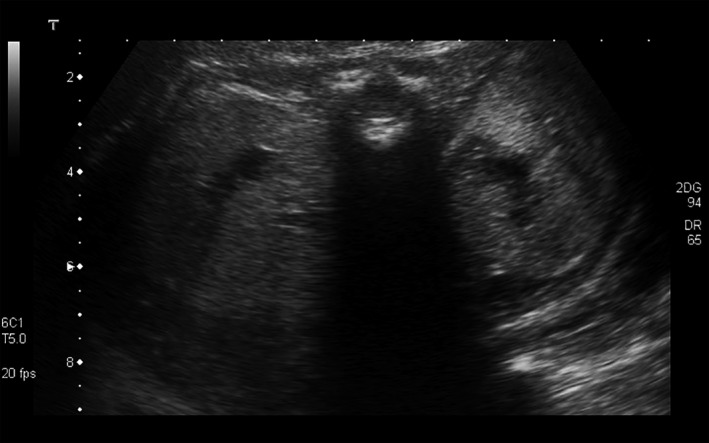
Bilateral enlarged hyperechogenic kidneys.

Here, we document a case of BBS diagnosed during the second trimester of pregnancy with prenatal suspicion presented by routine ultrasound examination findings of enlarged and hyperechogenic kidneys (Figs 1 and 2).

**Figure 3:**
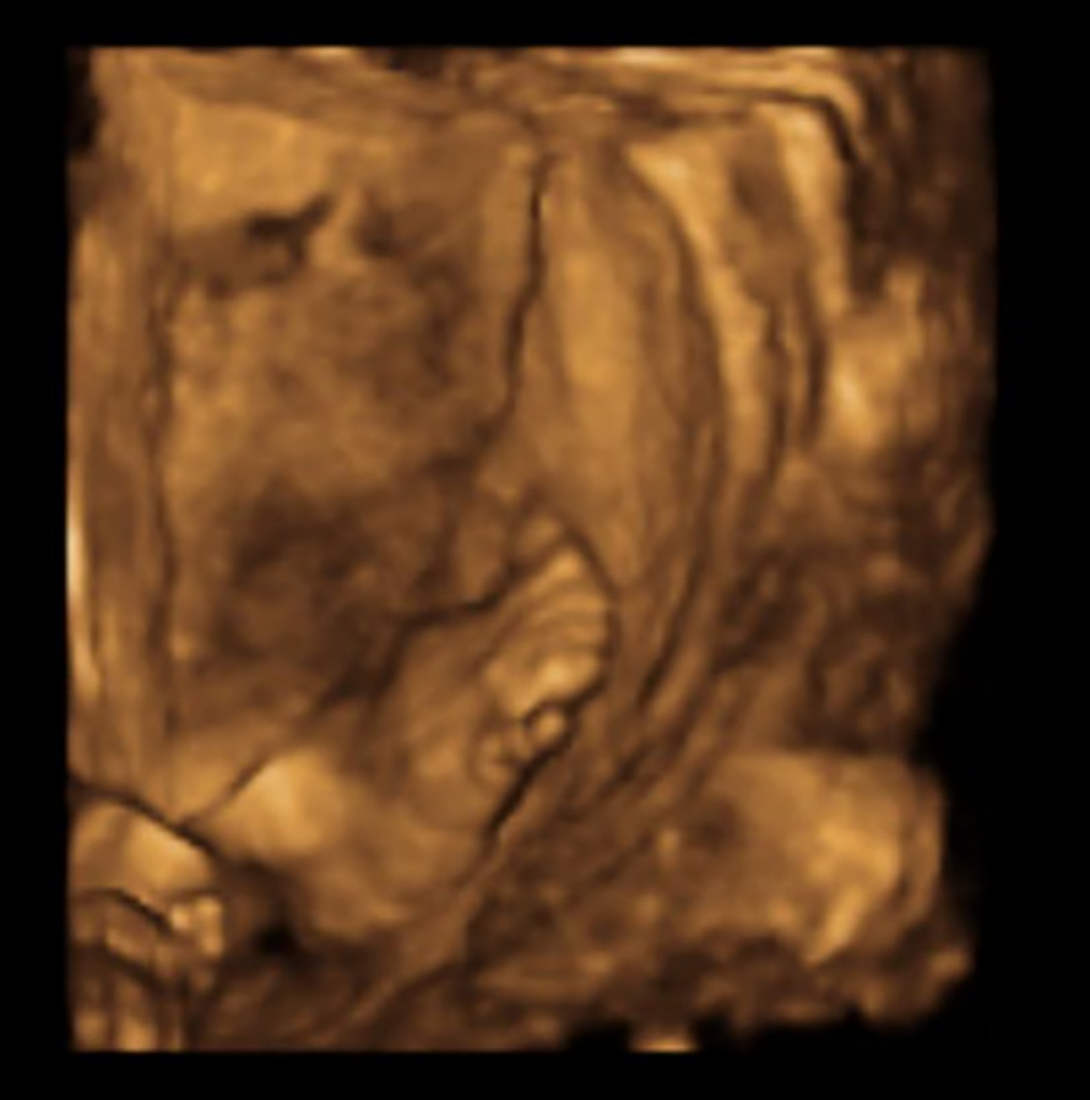
Unilateral right postaxial foot polydactyly.

Unilateral right postaxial foot polydactyly was also observed on the 20‐week scan with no other relevant findings (Fig. 3). The bladder size and amniotic fluid appeared normal until late pregnancy.

A prenatal ultrasound examination and assessment of fetal growth did not suggest any other relevant abnormalities during the third trimester of pregnancy.

## Investigations

Due to the suspicion of a genetic syndrome, an uncomplicated amniocentesis was performed at 20 + 6 weeks of gestation.

We performed next‐generation sequencing (NGS) of cultured amniotic fluid cells of a panel of the following 57 genes associated with Meckel‐Gruber syndrome and neuronal migration disorders: B9D1, B9D2, CC2DA, CEP290, MKS1, NPHP3, RPGRIP1L, TCTN2, TMEM216, TMEM23T, TMEM67, LV2982, AKT3, ALX4, ATP7A, ARX, CASK, CDK16, CDON, CHMP1A, EMX2, DKK1, DCX, DYNC1H1, EXOSC3, FLNA, GLI2, GPR56, HCCS, HEPACAM, IGBP1, LAMB1, L1CAM, PAFAH1B1, PTCH1, OCLN, PIK3R2, RARS2, RELN, RTTN, SEPSECS, SHH, SIX3, TGIF1, TSEN2, TSEN34, TSEN54, TUBA8, TUBB2B, TUBB3, TUBA1A, VRK1, YWHAE, ZIC1, ZIC2, ZIC3, ZIC4, LV3026, LV0912, and MKKS.

The regions to be studied were selected by probe hybridization and included exonic and adjacent intronic regions (±8 bp). These regions were subsequently amplified and paired‐end sequenced with an Illumina MiSeq platform. The read depth was between 519.00 x and 533.70 x, and the sequencing coverage was 97.9%.

The DNA sequence data were compared to the reference sequence by bioinformatic analysis, which defined as variants all alterations with >20 reads and a variant/read ratio >0.2. Pathogenic or possibly pathogenic variants were confirmed by Sanger sequencing. The variants of unknown significance were tested in silico (Alamut software V 2.0 141 boulevard de l'Yser76000 RouenFrance).

The genetic sequencing study showed that the fetus was a carrier of the McKusick–Kaufman syndrome (MKKS) gene mutation p.Val291Phe and a presumably pathogenic variant of the same gene, p.SEr236*, both in heterozygosis.

This result indicated that the fetus might be affected by either MKKS or BBS. Suspicion of the latter condition was supported by the absence of fetal cardiac abnormalities, the observation of renal pathology, and the presence of digital anomalies. However, an accurate diagnosis should be made in early childhood and with the onset of visual impairment in later childhood or teenage years.

A genetic investigation was also performed in both parents. The mother was found to be a carrier of the p.Ser236* mutation. The father appeared to be a carrier of Val291Phe and pGlu1985Asp variants in the CEP290 gene.

## Outcome and Follow‐up

Labor was induced at 37 weeks due to oligo‐anhydramnios and the suspicion of renal function deterioration. The following outcome occurred after an assisted vaginal delivery with forceps.

A 3480‐g male neonate, Apgar 6/8, pH 7.23, was born in our center. No resuscitation was required. The baby was admitted to the neonatal intensive care unit (NICU) due to hypoglycemia and for further study.

Abdominal distension and vomiting developed 24 h after birth. A simple abdominal X‐ray showed a markedly dilated bowel. A nasogastric tube was placed, and serial rectal irrigations were performed.

Fever and increased abdominal distension were evident 4 days after delivery. An emergent laparotomy was conducted. During the procedure, a sigmoid perforation closure, ileostomy, and biopsy were performed with no relevant complications.

Histologic evaluation of the biopsy demonstrated an absence of ganglion cells in the sigmoid submucosal and myenteric plexuses, which indicates a definitive finding of Hirschsprung disease.

The neonate was discharged from the NICU after one month and sent home with spontaneous diuresis of 2.9 cc/kg/h and a creatinine level of 0.69 mg/dL in the most recent blood test results. Further follow‐up is scheduled.

## Discussion

The most frequent genetically transmitted renal diseases that are evident during antenatal ultrasound examination are ARPKD and ADPKD.

In addition to these pathologies, autosomal dominant and autosomal recessive diseases, chromosomal aberrations, orofacial X‐linked syndrome, and VACTERL should be considered in the differential diagnosis 10.

Syndromes that present with hyperechoic kidneys and/or renal cysts are differentiated by their associated anomalies (Table 1).

**Table 1:** Characteristics and differential diagnosis of enlarged hyperechogenic kidneys and the associated syndromes

Syndrome	Transmission	Frequency	Findings
ADPKD	AD	1/1000	Enlarged hyperechogenic kidneys, increased CMD, normal amniotic fluid, uncommon associated malformations
ARPKD	AR	1/40,000	Enlarged hyperechogenic kidneys, absence of CMD, oligohydramnios, uncommon associated malformations
Meckel‐Gruber	AR	Rare	Medullary cystic dysplasia, severe oligohydramnios, CNS anomaly, polydactyly
Beckwith Wiedemann	Imprinting disorder	1/14,000	Macroglossia, macrosomia, omphalocele, hemihyperplasia, renal medullary dysplasia, polyhydramnios
Bardet–Biedl	AR	1/125–160,000	Enlarged hyperechogenic kidneys (30–100%), absence of CMD, digital anomalies
McKusick–Kaufman	AR	1/10,000	Postaxial polydactyly, congenital heart disease (CHD), hydrometrocolpos in females, genital malformations in males
Ivemark II	AR	Rare	Asplenia–polysplenia, cystic liver, kidney and pancreas
Jarcho‐Levin	AR	Rare	Spondylothoracic dysplasia, generally lethal
Beemer	AR	Rare	Narrow ribs, micromelia, multiple anomalies of major organs, polydactyly
Oral facial digital syndrome type 1 (OFD1)	X‐linked recessive	1/50,000–1/250,000	Cleft lip or palate, hypertelorism, micrognathia, brachydactyly, syndactyly, preaxial or postaxial polydactyly, intracerebral cysts, agenesis of the corpus callosum, Dandy‐Walker malformation
Short rib‐polydactyly syndromes	AR	Rare	Cleft lip or palate, narrow thorax with short ribs, shortening of all bones, polydactyly, small cerebellar vermis
Tuberous sclerosis complex	AD/de novo mutation	1/5800	Angiofibromas, ungual fibromas, cortical tubers, angiolipomas in the kidney, cardiac rhabdomyomas
Jeune syndrome	AR	1/100,000–130,000	Skeletal dysplasia, narrow thorax with short ribs, shortening of long bones, polydactyly, brachydactyly
Zellweger syndrome	AR	1/50,000–100,000	Intrauterine growth restriction, muscular hypotonia, increased nuchal translucency, abnormal head shape, micrognathia, hydrocephalus, ventriculomegaly, agenesis, or hypoplasia of the corpus callosum
Trisomy 13	Chromosomal aberration	1/1000	Omphalocoele, umbilical hernia, genitourinary abnormalities, superficial hemangiomas, polydactyly, club foot (congenital talipes equinovarus), congenital heart defects
Trisomy 18	Chromosomal aberration	1/5500	Intrauterine growth restriction, hypertonia, prominent occiput, micrognathia, short sternum, horseshoe kidney, flexed fingers, congenital heart defects

ARPKD, autosomal recessive polycystic kidney disease; ADPKD, autosomal dominant polycystic kidney disease; AD, autosomal dominant; AR, autosomal recessive; CMD, corticomedullary differentiation; CNS, central nervous system.

The diagnostic work‐up of fetal hyperechogenic kidneys by ultrasound should include a thorough fetal anatomic examination, review of the family's genetic history, and ultrasound examination of the kidneys of the parents. The increased risk of an abnormal fetal karyotype should be discussed, and prenatal testing offered if nonrenal malformations are discovered.

Prenatal suspicion of BBS/MKKS can arise from the findings of large hyperechogenic kidneys and polydactyly observed at the time of routine ultrasound examination. This finding presents a difficult diagnosis, especially when the amniotic fluid volume is normal. The normal renal cortex is equally echogenic to the liver or spleen, whereas the medulla is hypoechogenic. By definition, a hyperechogenic kidney has a cortex that is hyperechogenic to either the liver or the spleen 11.

Ultrasound examination of fetuses with hyperechoic kidneys includes assessment of:

- Amniotic fluid volume
- Kidney and bladder size
- Corticomedullary differentiation
- Renal echogenicity
- Location of renal cysts

A family history and genetic survey are required. Often, the key to diagnosis will be the demonstration of associated malformations.

The clinical diagnostic criteria for BBS are subdivided into primary and secondary criteria. Either four primary or three primary and two secondary criteria are satisfactory for a clinical diagnosis of BBS 12.

Regarding Hirschsprung disease, it is known that 30% of cases are syndromic. A causative gene has been identified in eight Mendelian conditions, including congenital central hypoventilation, BBS, Smith–Lemli–Opitz syndrome, Mowat–Wilson syndrome, Shah‐Waardenburg syndrome, cartilage‐hair hypoplasia, hydrocephalus due to congenital stenosis of the aqueduct of Sylvius, and Goldberg–Shprintzen syndrome. Wide variation occurs in the severity and segment involvement of Hirschsprung disease in patients with BBS. However, the mechanism of this phenomenon remains unclear, as few cases have been reported in the literature 13.

It is essential to note the age dependency of some of the more important diagnostic findings, including the onset of obesity after infancy and developmental differences and visual impairment later in childhood (Table 2).

**Table 2:** Major and secondary clinical diagnostic criteria for BBS

Major criteria	Secondary criteria
Rod‐cone dystrophy	Strabismus, cataracts or astigmatism
Obesity	Speech disorder or speech delay
Polydactyly	Brachydactyly or syndactyly
Renal anomalies	Developmental delay
Learning difficulties	Ataxia and/or poor coordination
Hypogenitalism in males	Mild spasticity
	Polyuria and polydipsia
	Diabetes mellitus
	Dental crowding or hypodontia
	Left ventricular hypertrophy or congenital heart disease
	Hepatic fibrosis

Retinitis pigmentosa, mental retardation, and obesity do not develop in BBS patients until at least 5 years of age. Therefore, children initially diagnosed with MKKS should be followed up for further re‐evaluation.

## Authorship

SGTL: performed literature review. VBA: performed literature review. MOC: performed genetic counseling. AAM: performed image collection. VOA: performed literature review. RPFP: performed literature review. JDLL: performed literature review. FGA: performed literature review and perinatal counseling.

## Conflict of Interests

The authors have no conflict of interests to declare.
